# The MRX Complex Plays Multiple Functions in Resection of Yku- and Rif2-Protected DNA Ends

**DOI:** 10.1371/journal.pone.0014142

**Published:** 2010-11-30

**Authors:** Diego Bonetti, Michela Clerici, Nicola Manfrini, Giovanna Lucchini, Maria Pia Longhese

**Affiliations:** Dipartimento di Biotecnologie e Bioscienze, Università di Milano-Bicocca, Milano, Italy; University of Medicine and Dentistry of New Jersey, United States of America

## Abstract

The ends of both double-strand breaks (DSBs) and telomeres undergo tightly regulated 5′ to 3′ resection. Resection of DNA ends, which is specifically inhibited during the G1 cell cycle phase, requires the MRX complex, Sae2, Sgs1 and Exo1. Moreover, it is negatively regulated by the non-homologous end-joining component Yku and the telomeric protein Rif2. Here, we investigate the nuclease activities that are inhibited at DNA ends by Rif2 and Yku in G1 versus G2 by using an inducible short telomere assay. We show that, in the absence of the protective function of Rif2, resection in G1 depends primarily on MRX nuclease activity and Sae2, whereas Exo1 and Sgs1 bypass the requirement of MRX nuclease activity only if Yku is absent. In contrast, Yku-mediated inhibition is relieved in G2, where resection depends on Mre11 nuclease activity, Exo1 and, to a minor extent, Sgs1. Furthermore, Exo1 compensates for a defective MRX nuclease activity more efficiently in the absence than in the presence of Rif2, suggesting that Rif2 inhibits not only MRX but also Exo1. Notably, the presence of MRX, but not its nuclease activity, is required and sufficient to override Yku-mediated inhibition of Exo1 in G2, whereas it is required but not sufficient in G1. Finally, the integrity of MRX is also necessary to promote Exo1- and Sgs1-dependent resection, possibly by facilitating Exo1 and Sgs1 recruitment to DNA ends. Thus, resection of DNA ends that are protected by Yku and Rif2 involves multiple functions of the MRX complex that do not necessarily require its nuclease activity.

## Introduction

Intrachromosomal DNA double-strand breaks (DSBs) are among the most deleterious chromosomal lesions that can occur either spontaneously or after exposure to DNA damaging agents. Depending on the cell cycle phase at which DSBs are detected and on the nature of the DSB ends, homologous recombination (HR) or non-homologous end-joining (NHEJ) are used to repair them (reviewed in [1]). Furthermore, DSBs also elicit a checkpoint response, which coordinate cell cycle progression with DNA repair capacity (reviewed in [2]–[4]).

Eukaryotic cells have to deal also with the natural ends of linear chromosomes, which are structurally similar to DSB ends but must be protected from fusion, degradation and recognition by the checkpoint machinery (reviewed in [5]–[7]). This protection depends on chromosomal end packaging into nucleoprotein complexes called telomeres and it is crucial not only for genome integrity and suppression of tumorigenesis, but also for cell viability (reviewed in [8]).

Telomeric DNA consists of short tandem DNA repeats that are G-rich in the 3′-strand (3′ G-strand), which protrudes beyond the 5′-end, forming a single-stranded overhang (G tail) (reviewed in [9], [10]). Both double-stranded and single-stranded telomeric DNA regions are specifically bound by proteins that regulate telomeric DNA replication by telomerase. In *Saccharomyces cerevisiae*, Cdc13 binds the single-stranded G tails and is necessary for telomerase-dependent telomere elongation, because it mediates telomerase recruitment [11]. Conversely, telomerase action is negatively regulated by Rap1, Rif1 and Rif2 [12], [13], which bind telomeric double-stranded DNA and also inhibit NHEJ at telomeres [14].

Both intrachromosomal DSBs and telomeres undergo 5′ to 3′ nucleolytic degradation of DNA ends, a process known as resection (reviewed in [15]). The subsequent generation of 3′-ended single-strand DNA (ssDNA) channels DSB repair into HR, whereas it ensures telomere replication by providing a substrate for telomerase. It is thought that cyclin-dependent kinase activity (Cdk1 in *S. cerevisiae*) enhances resection of a DSB by phosphorylating Sae2 [16], [17], which co-operates with the Mre11/Rad50/Xrs2 (MRX) complex in the initial resection of the 5′ strand, possibly through an endonucleolytic cleavage of 50–100 nt [18], [19]. The resulting partially resected 5′ DNA end can be further processed by either Exo1 or Sgs1 and Dna2 [18]–[20].

Cdk1 activity is required to generate ssDNA also at telomeres [21], [22], and this requirement coincides in time with telomere replication by telomerase [23]. The generation of telomeric ssDNA appears to have similar requirements in terms of nucleases and Cdk1-dependent Sae2 phosphorylation as the processing events at a DSB [24]–[26]. In fact, by using a system in which an HO endonuclease cleavage site is adjacent to a short telomere seed sequence (HO-induced telomere), it has been shown that MRX and Sae2 are involved in resection of the HO-induced telomere, with MRX playing a major function [24], [26]. When Sae2 is absent, Exo1 and Sgs1 in turn can resect the HO-induced telomere, with Sgs1 acting in conjunction with Dna2 [26].

The MRX complex exhibits 3′-5′ exonuclease and endonuclease activities [27], [28], which reside in the Mre11 subunit. Also Sae2 displays ssDNA endonuclease activity [29], raising the possibility that Sae2 may act either as a regulator of Mre11 nuclease activity or as a nuclease. In budding yeast, Mre11 nuclease activity is required for processing of meiotic DSBs and DNA hairpins, but is dispensable for resection of DNA ends generated by the HO endonuclease [30]–[33]. In fact, *mre11* nuclease defective mutants show only mild sensitivity to DNA damaging agents and weak resection defects when compared to *mre11Δ* cells. This finding suggests that MRX has a role in resection independently of its nuclease activity and this function cannot be compensated by the activity of other nucleases.

Resection is less extensive at telomeric ends than at intrachromosomal DSB ends, and this limitation depends on proteins that counteract nuclease action. In particular, inactivation of Cdc13 leads to accumulation of ssDNA regions at both telomeric and sub-telomeric *S. cerevisiae* DNA sequences [34]–[36]. Furthermore, the heterodimeric Yku complex (Yku70-Yku80) contributes to protect telomeres from degradation [37]–[40] and this protective function becomes apparent in G1 [41], [42]. Finally, inactivation of the shelterin-like proteins Rif2 and Rap1 leads to telomere nucleolytic degradation in G1 and enhances it in G2 [41], [42]. Telomeric ssDNA generation is increased to the same extent in the absence of Rif2 or Rap1 C-terminus [41], suggesting that the inhibitory effect exerted by Rap1 is likely mediated by Rif2, whose recruitment to telomeres depends on Rap1 C-terminal domain [43]. Exo1 is primarily responsible for telomere resection in *yku70Δ* G1 cells [41], [42], whereas the absence of MRX prevents telomeric ssDNA generation in *rif2Δ* cells [41]. Recruitment of MRX at telomeres is enhanced in cells lacking either Rif2 or the Rap1 C-terminal domain [41], [44], suggesting that Rap1 and Rif2 can prevent MRX action by inhibiting MRX association to telomeric ends. Thus, while Yku protects telomeres from Exo1 action, Rap1 and Rif2 prevent degradation of telomeres by inhibiting MRX loading onto their ends. However, given that MRX has a role in resection independently of its nuclease activity, it is currently unknown the nature of the nuclease that is inhibited by Rif2 and how Rif2 and Yku coordinate their functions during the cell cycle.

By using an inducible short telomere assay in cells lacking the protective function of Rif2, we show that resection in G1 requires primarily MRX nuclease activity and Sae2. On the other hand, Exo1 and Sgs1 compensate for defective MRX nuclease activity in G1 cells in the absence of Yku, suggesting that Yku inhibits not only Exo1 but also Sgs1 in this cell cycle phase. Furthermore, Yku-mediated inhibition of Exo1 and Sgs1 is relieved in G2 cells, where resection depends on MRX nuclease activity, as well as on Exo1 and, to a minor extent, Sgs1. While Yku is able to prevent Exo1 action in G1 either in the presence or in the absence of MRX, Yku-mediated inhibition of Exo1 in G2 becomes apparent only when MRX is physically lacking, and not when an Mre11 nuclease-defective variant is present. These findings suggest that binding of MRX to DNA ends is required and sufficient for Yku removal in G2, whereas it is required but not sufficient in G1. Finally, MRX is required to allow Sgs1-mediated resection and to increase the efficiency of Exo1 resection activity independently of Mre11 nuclease activity.

## Results

### Resection in G1 in the absence of Rif2 depends primarily on Mre11 nuclease activity and Sae2

**Figure 1:**
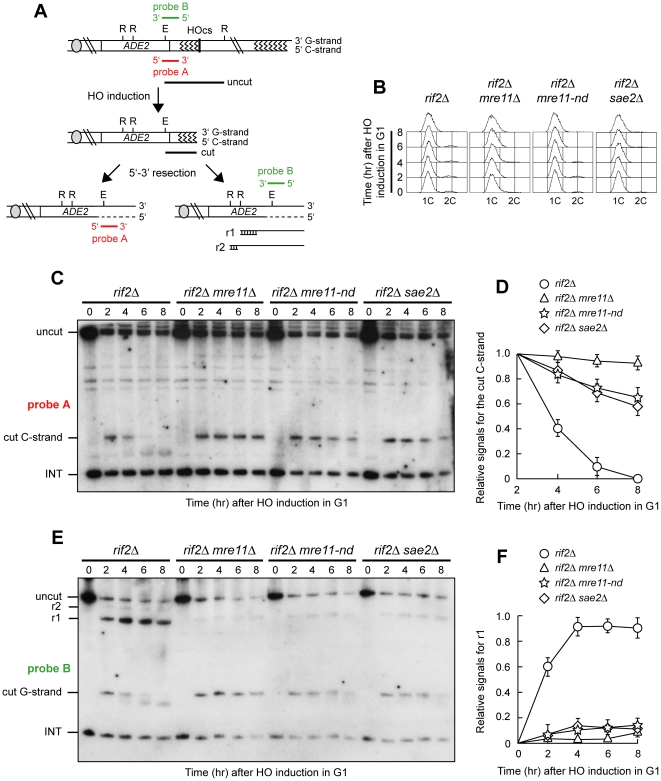
The lack of either Sae2 or Mre11 nuclease activity severely impairs DNA end resection in G1 cells lacking Rif2. (A) The HO-induced telomere system. See text for details. The TG repeat sequences are indicated by zigzag lines. Resection of the 5′ C-strand is indicated by dashed lines. The two single-stranded riboprobes that anneal to either the 5′ C-strand (probe A) or the 3′ G-strand (probe B) to a site located 212 bp from the HO cutting site are indicated. Both probes also detects a 138 nt fragment from the *ade2-101* locus on Chr. XV (INT), which serves as internal loading control. (B–F) HO expression was induced at time zero by galactose addition to α-factor-arrested cell cultures with the indicated genotypes that were then kept arrested in G1. (B) FACS analysis of DNA content. (C) RsaI- and EcoRV-digested genomic DNA was hybridized with probe A. Degradation of the 5′ C-strand leads to the disappearance of the 166 nt signal (cut C-strand) generated by this probe. (D) Densitometric analysis. Plotted values are the mean value ±SD from three independent experiments as in (C). (E) The same RsaI- and EcoRV-digested genomic DNA analyzed in (C) was hybridized with probe B. Degradation of the 5′ C-strand leads to the conversion of the 3′ cut G-strand 166 nt fragment into the slower migrating r1 and r2 DNA fragments described in (A). (F) Densitometric analysis. Plotted values are the mean value ±SD from three independent experiments as in (E).

Cells devoid of Rif2 display accumulation of ssDNA in G1 at both HO-induced and native telomeres [41]. The lack of MRX due to deletion of *MRE11* abolishes ssDNA generation at telomeres in *rif2Δ* cells, indicating that Rif2 counteracts the action of MRX [41]. As the lack of MRX causes more severe resection defects than the lack of its nuclease activity [30], [33], we asked whether the latter was required for DNA end resection in *rif2Δ* G1 cells. To this end, we used a system originally developed to examine de novo telomere synthesis in the budding yeast *S. cerevisiae* [24]. Briefly, a galactose-inducible version of the HO endonuclease gene is carried by a yeast strain containing an HO endonuclease cleavage site adjacent to an 81-base pair TG repeat sequence that is inserted at the *ADH4* locus, 15 kb from the left telomere of chromosome VII (Figure 1A). Upon cleavage by HO, this short TG sequence generates an HO-induced telomere that is elongated by telomerase [24]. Length changes of either the 5′ C-strand or the 3′ G-strand of this newly created telomere can be monitored by using two single-stranded riboprobes (probes A and B in Figure 1A) that recognize the 5′ C-strand or the 3′ G-strand, respectively, by annealing to a DNA region located 212 bp from the HO cleavage site (Figure 1A). Upon digestion of genomic DNA with EcoRV (E) and RsaI (R) restriction enzymes, both probes reveal an uncut 390 nt DNA fragment (uncut), which is converted by HO cleavage into a 166 nt fragment (cut) that can be detected by both probe A (5′ C-strand) and probe B (3′ G-strand) (Figure 1A). Degradation of the 5′ C-strand leads to disappearance of the probe A signal as resection proceeds beyond the hybridization region. Furthermore, progressive 5′ C-strand resection eliminates the cutting sites for the EcoRV and RsaI restriction enzymes, thus resulting in the conversion of the 166 nt EcoRV-HO 3′ G-strand fragment into the slower migrating DNA fragments r1 (304 nt) and r2 (346 nt) detected by probe B (Figure 1A).

To determine the contribution of MRX nuclease activity in resecting the HO-induced telomere in *rif2Δ* G1 cells, we used an allele of *MRE11* (*mre11-H125N*, here referred to as *mre11-nd*) encoding a protein that lacks both endonuclease and exonuclease activities but does not affect either the stability of the MRX complex [45] or its ability to bind DNA ends [46]. After HO-induction in G1-arrested *rif2Δ* cells (Figure 1B), the 5′ C-strand of the HO-induced telomere was analyzed with its complementary probe A in EcoRV and RsaI double-digested genomic DNA (Figure 1C and 1D). As expected from the finding that Rif2 prevents MRX function [41], the predicted EcoRV-HO band (166 bp; cut C-strand) corresponding to the 5′ C-rich strand of the HO-induced telomere remained stable in G1-arrested *rif2Δ mre11Δ* cells, whereas its amount progressively decreased in *rif2Δ* cells (Figure 1C and 1D). DNA degradation in *rif2Δ* G1 cells depends mainly on Mre11 nuclease activity. In fact, the 5′ C-strand signal decreased only slightly in G1-arrested *rif2Δ mre11-nd* cells compared to similarly treated *rif2Δ* cells (Figure 1C and 1D). Furthermore, the very limited resection of the 5′ C-strand in *rif2Δ mre11-nd* G1 cells did not proceed beyond the EcoRV site located 166 bp from the HO site. In fact, analysis of the same DNA samples with the 3′ G-strand complementary probe B did not reveal any accumulation of r1 and r2 resection products in *rif2Δ mre11-nd* G1 cells, while they were detectable in *rif2Δ* cells (Figure 1E and 1F). Consistent with previous observations [24], [41], 3′ G-strand length of the HO-induced telomere decreased by ∼10 nucleotides in *rif2Δ* cells (Figure 1E), but the functional role of this degradation is unknown. Thus, Mre11 nuclease activity is required for the nucleolytic processing of the HO-induced telomere in G1 cells lacking Rif2.

MRX is thought to act in concert with Sae2 with respect to DNA end resection. In fact, *MRE11* and *SAE2* belong to the same epistasis group and *mre11-nd* mutants display a similar phenotype to *sae2Δ* mutants [31], [32], [47]. Indeed, Sae2 turned out to be important for telomere resection in *rif2Δ* G1 cells, as 5′ C-strand degradation was severely impaired in G1-arrested *rif2Δ sae2Δ* cells (Figure 1C and 1D), which also failed to generate r1 and r2 resection products (Figure 1E and 1F). Altogether, these data indicate that MRX nuclease activity and Sae2 are important for resecting DNA ends in G1 when the protective function of Rif2 is missing.

### Exo1 and Sgs1 are dispensable for resection in G1 in the absence of Rif2

**Figure 2:**
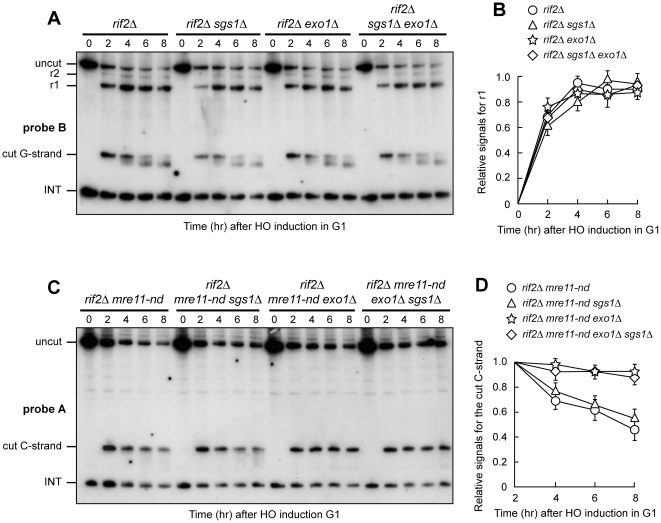
Exo1 and Sgs1 are dispensable for resection in *rif2Δ* G1 cells. HO expression was induced at time zero by galactose addition to α-factor-arrested cell cultures that were then kept arrested in G1. (A) RsaI- and EcoRV-digested genomic DNA was hybridized with probe B as in Figure 1E. (B) Densitometric analysis. Plotted values are the mean value ±SD from three independent experiments as in (A). (C) RsaI- and EcoRV-digested genomic DNA was hybridized with probe A as in Figure 1C. (D) Densitometric analysis. Plotted values are the mean value ±SD from three independent experiments as in (C). The cell cycle arrest in G1 of all cell cultures was confirmed by FACS analysis at each time point (data not shown).

Resection of an HO-induced telomere in G2 was shown to require both Sgs1 and Exo1, as it was severely affected by their concomitant absence, although the lack of each single protein did not cause resection defects [26]. Thus, we asked whether Rif2 protects telomeric ends in G1 not only from MRX but also from Exo1 and/or Sgs1. To this end, we analyzed 3′ G-strand of the HO-induced telomere in *rif2Δ* cells lacking Exo1 and/or Sgs1 by using the G-strand complementary probe B (Figure 2A and 2B). The amount of EcoRV-HO fragment (cut G-strand) decreased with similar kinetics in G1-arrested *rif2Δ*, *rif2Δ sgs1Δ*, *rif2Δ exo1Δ* and *rif2Δ sgs1Δ exo1Δ* cells, which showed similar amount of r1 and r2 resection products (Figure 2A and 2B). As expected, when the same DNA samples were analysed with probe A, C-strand degradation occurred with similar kinetics in all cell cultures (data not shown). Thus, the lack of Exo1 or Sgs1 or both does not affect 5′ C-strand degradation in G1-arrested *rif2Δ* cells when MRX is functional. Similar to what we observed in *rif2Δ* cells (Figure 1E), 3′ G-strand length of the HO-induced telomere decreased by ∼10 nucleotides in all the above cell cultures (Figure 2A).

We also asked whether Exo1 and/or Sgs1 are responsible for the residual resection detected in G1-arrested *rif2Δ mre11-nd* cells. As G-strand analysis failed to detect accumulation of resection products in *rif2Δ mre11-nd* cells (Figure 1E and 1F), we analyzed C-strand degradation by using probe A. As shown in Figure 2C and 2D, the amount of the 5′ C strand signal decreased with similar kinetics in both *rif2Δ mre11-nd* and *rif2Δ mre11-nd sgs1Δ* cells, whereas it remained constant in both *rif2Δ mre11-nd exo1Δ* and *rif2Δ mre11-nd exo1Δ sgs1Δ* cells. Thus, only Exo1 participates in resection in G1 when Mre11 nuclease activity is defective, although it does not bypass MRX requirement for efficient DNA end processing. The finding that 5′ C-strand was not degraded in G1-arrested *rif2Δ mre11Δ* cells (Figure 1C and 1D), whereas a residual Exo1-dependent 5′ C-strand degradation was detectable in G1-arrested *rif2Δ mre11-nd* cells (Figure 2C and 2D) suggests that Exo1-dependent resection in G1 likely requires the presence of MRX, but not Mre11 nuclease activity.

### Exo1 is more important for resection in G2 than in G1 when Mre11 nuclease activity is defective

**Figure 3:**
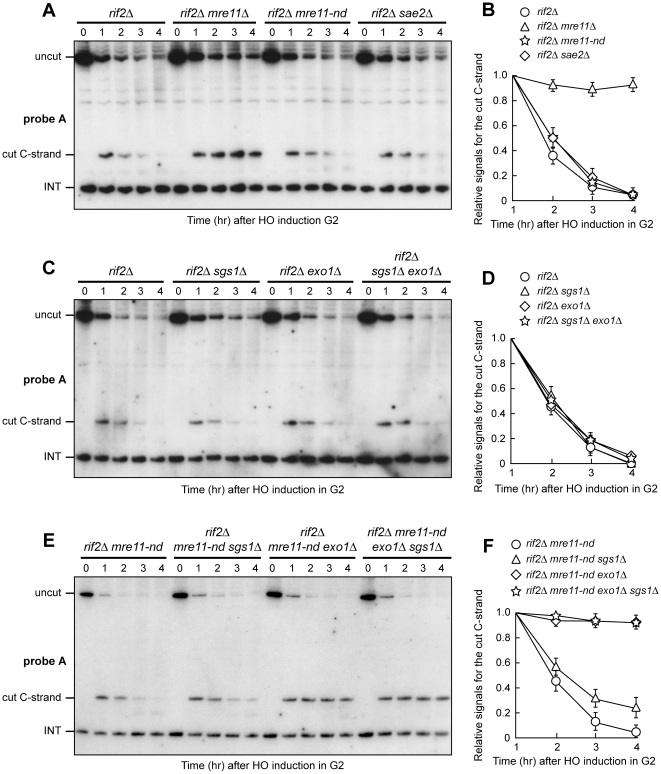
Nuclease requirements for DNA end resection in G2 cells lacking Rif2. HO expression was induced at time zero by galactose addition to nocodazole-arrested cell cultures that were then kept arrested in G2. (A, C, E) RsaI- and EcoRV-digested genomic DNA was hybridized with probe A as described in Figure 1C. (B) Densitometric analysis. Plotted values are the mean value ±SD from three independent experiments as in (A). (D) Densitometric analysis. Plotted values are the mean value ±SD from three independent experiments as in (C). (F) Densitometric analysis. Plotted values are the mean value ±SD from three independent experiments as in (E). The cell cycle arrest in G2 of all cell cultures was confirmed by FACS analysis at each time point (data not shown).

The lack of Rif2 enhances the efficiency of resection of an HO-induced telomere even in G2 [41]. Similar to what we observed in G1, the lack of MRX abolishes resection of the HO-induced telomere in *rif2Δ* G2-arrested cells, as the 5′ C-strand signal remained stable in G2-arrested *rif2Δ mre11Δ* cells (Figure 3A and B). However, while MRX nuclease activity and Sae2 are important for DNA end resection in *rif2Δ* G1 cells, they become dispensable in G2. In fact, degradation of the 5′ C-strand was not impaired in either *rif2Δ mre11-nd* or *rif2Δ sae2Δ* G2-arrested cells (Figure 3A and 3B). Disappearance of the 5′ C-strand signal in all these cell cultures was due to DNA degradation and not to elongation by the coordinated action of telomerase and lagging strand DNA synthesis, as we observed ssDNA generation also in *rif2Δ*, *rif2Δ mre11-nd* and *rif2Δ sae2Δ* G2 cells lacking the catalytic subunit of telomerase (data not shown).

The 5′ C-strand signal decreased with similar kinetics also in G2-arrested *rif2Δ sgs1Δ*, *rif2Δ exo1Δ* and *rif2Δ sgs1Δ exo1Δ* cells (Figure 3C and 3D). However, 5′ C-strand degradation was undetectable in G2-arrested *rif2Δ mre11-nd exo1Δ* cells (Figure 3E and 3F), indicating that Exo1 and MRX nuclease activity provide redundant activities to resect the HO-induced telomere in *rif2Δ* G2 cells. As Mre11 nuclease activity is important for resection in G1 but dispensable in G2, these findings indicate that Exo1 resects DNA ends more efficiently in G2 than in G1.

Degradation of the 5′ C-strand was slightly affected also in G2-arrested *rif2Δ mre11-nd sgs1Δ* cells (Figure 3E and 3F), indicating that also Sgs1 contributes to resection in G2 when MRX nuclease activity is defective, although with a minor role compared to Exo1.

### Yku inhibits Exo1- and Sgs1-dependent resection in G1 when Mre11 nuclease activity is defective

**Figure 4:**
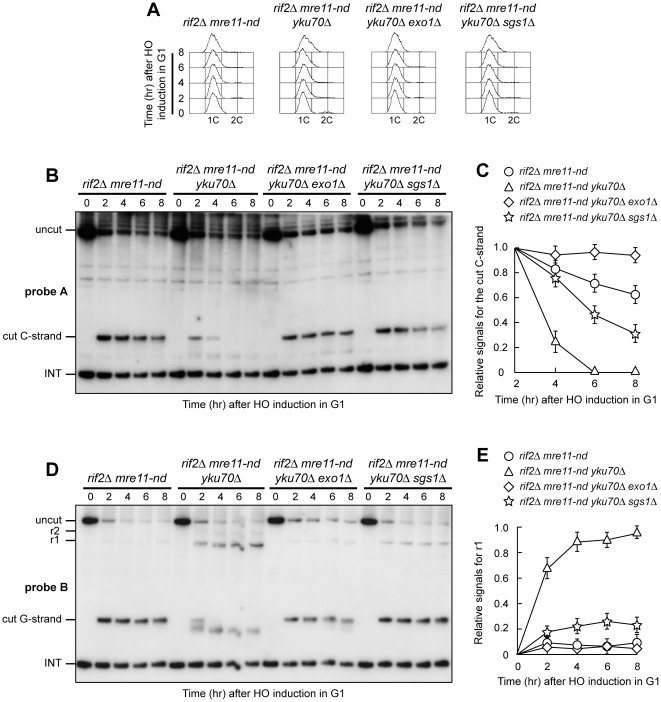
Yku absence allows Exo1- and Sgs1-dependent resection in *rif2Δ* G1 cells with nuclease-defective MRX. HO expression was induced at time zero by galactose addition to α-factor-arrested cells that were then kept arrested in G1. (A) FACS analysis of DNA content. (B) RsaI- and EcoRV-digested genomic DNA was hybridized with probe A as in Figure 1C. (C) Densitometric analysis. Plotted values are the mean value ±SD from three independent experiments as in (B). (D) The same RsaI- and EcoRV-digested genomic DNA analyzed in (B) was hybridized with probe B as in Figure 1E. (E) Densitometric analysis. Plotted values are the mean value ±SD from three independent experiments as in (D).

Yku is known to protect both native and HO-induced telomeres from degradation in G1 by preventing Exo1 function [41], [42]. Furthermore, the lack of Yku was shown to suppress in an Exo1-dependent manner the hypersensitivity to DNA damaging agents of *S. pombe* cells lacking the Sae2 ortholog Ctp1 [48]. Thus, one possibility is that this Yku-mediated inhibition can account for the inability of Exo1 to fully compensate for a defective Mre11 nuclease activity in G1. If this were the case, the lack of Yku should restore Exo1-dependent resection in G1-arrested *rif2Δ mre11-nd* cells (Figure 4A). Indeed, degradation of the 5′ C-strand, which was severely impaired in G1-arrested *rif2Δ mre11-nd*, took place in *rif2Δ mre11-nd yku70Δ* G1 cells (Figure 4B and 4C). Furthermore, *rif2Δ mre11-nd yku70Δ* G1 cells formed G-strand r1 resection products that were not detectable in similarly treated *rif2Δ mre11-nd* cells (Figure 4D and 4E). This ssDNA generation was Exo1-dependent, as 5′ C-strand degradation was undetectable in *rif2Δ mre11-nd yku70Δ exo1Δ* G1 cells (Figure 4B and 4C), which also failed to accumulate G-strand resection products (Figure 4D and 4E). Thus, the lack of Yku allows Exo1 to resect the HO-induced telomere in G1 cells when Rif2 is absent, indicating that Yku counteracts Exo1 resection function in G1 even in the absence of Rif2.

Interestingly, we found that degradation of the HO-induced telomere in *rif2Δ mre11-nd yku70Δ* G1-arrested cells depends partially also on Sgs1, as C-strand degradation was impaired in *rif2Δ mre11-nd yku70Δ sgs1Δ* G1 cells compared to *rif2Δ mre11-nd yku70Δ* (Figure 4B and 4C). Furthermore, *rif2Δ mre11-nd yku70Δ sgs1Δ* G1 cells also showed a lower amount of r1 resection product than *rif2Δ mre11-nd yku70Δ* cells (Figure 4D and 4E). Because the lack of Sgs1 does not affect resection in G1-arrested *rif2Δ mre11-nd* cells when Yku is functional (Figure 2C and 2D), this finding suggests that Yku protects the HO-induced telomere in G1 not only from Exo1, but also from Sgs1.

### Rif2 prevents Exo1-dependent resection in G2

**Figure 5:**
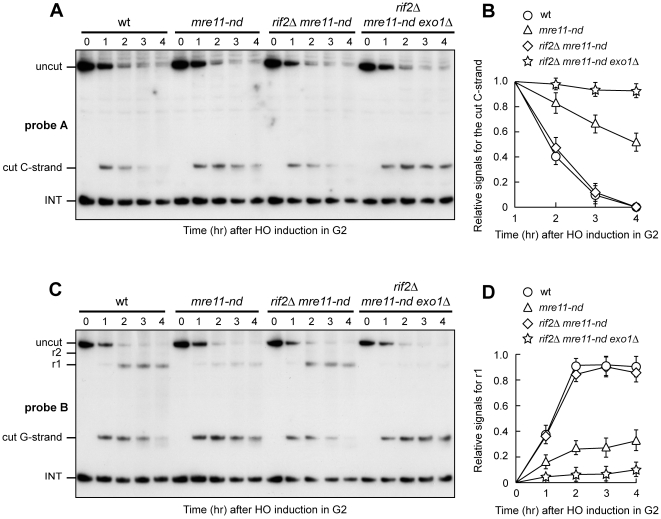
The lack of Rif2 enhances Exo1-dependent resection in *mre11-nd* G2 cells. HO expression was induced at time zero by galactose addition to nocodazole-arrested cells that were then kept arrested in G2. (A) RsaI- and EcoRV-digested genomic DNA was hybridized with probe A as in Figure 1C. (B) Densitometric analysis. Plotted values are the mean value ±SD from three independent experiments as in (A). (C) The same RsaI- and EcoRV-digested genomic DNA analyzed in (A) was hybridized with probe B as described in Figure 1E. (D) Densitometric analysis. Plotted values are the mean value ±SD from three independent experiments as in (C). The cell cycle arrest in G2 of all cell cultures was confirmed by FACS analysis at each time point (data not shown).

Exo1 compensates for the lack of Mre11 nuclease activity more efficiently than Sgs1 when Rif2 is absent. In fact, while the lack of Exo1 abolished resection in both G1- and G2-arrested *rif2Δ mre11-nd* cells, the lack of Sgs1 had little or no effect (Figures 2 and 3). Since Exo1 and Sgs1 play redundant functions in resection when Rif2 is functional [26], one possibility is that Rif2 counteracts Exo1 resection activity. This inhibitory function of Rif2 on Exo1 could become apparent only in G2, because the Yku-mediated block on Exo1 is relieved in this cell cycle phase. As Exo1 and MRX nuclease activity play redundant functions in G2, we then asked whether Rif2 removal results in increased amounts of ssDNA at the HO-induced telomere in G2-arrested *mre11-nd* cells. We found that *mre11-nd* cells degraded the 5′ C-strand less efficiently than *rif2Δ mre11-nd* G2 cells (Figure 5A and 5B). Furthermore, the amount of r1 and r2 resection products was lower in *mre11-nd* than in *rif2Δ mre11-nd* G2 cells (Figure 5C and 5D). Generation of ssDNA in *rif2Δ mre11-nd* cells occurred in an Exo1-dependent manner, as 5′ C-strand degradation (Figure 5A and 5B) and generation of G-strand resection products (Figure 5C and 5D) were undetectable in G2-arrested *rif2Δ mre11-nd exo1Δ* cells. Thus, Rif2 appears to prevent Exo1 function.

### MRX binding to DNA ends relieves Yku-mediated inhibition of Exo1 and increases the efficiency of Exo1-dependent resection

**Figure 6:**
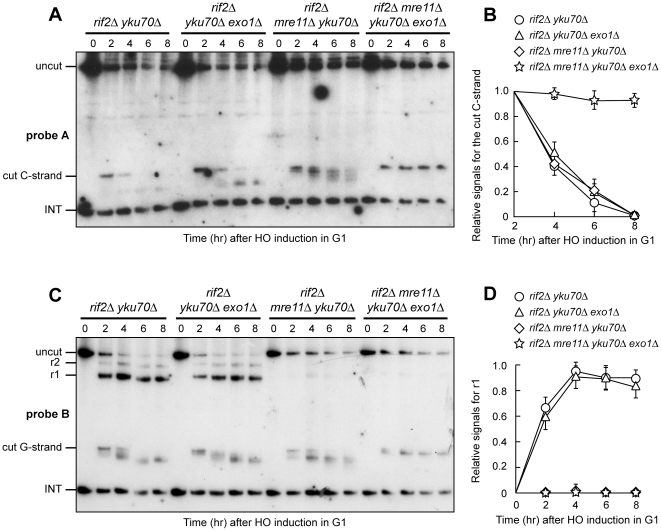
The lack of Yku allows Exo1-dependent resection in *rif2Δ* G1 cells lacking MRX. HO expression was induced at time zero by galactose addition to α-factor-arrested cells that were then kept arrested in G1. (A) RsaI- and EcoRV-digested genomic DNA was hybridized with probe A as in Figure 1C. (B) Densitometric analysis. Plotted values are the mean value ±SD from three independent experiments as in (A). (C) The same RsaI- and EcoRV-digested genomic DNA analyzed in (A) was hybridized with probe B as described in Figure 1E. (D) Densitometric analysis. Plotted values are the mean value ±SD from three independent experiments as in (C). The cell cycle arrest in G1 of all cell cultures was confirmed by FACS analysis at each time point (data not shown).

**Figure 7:**
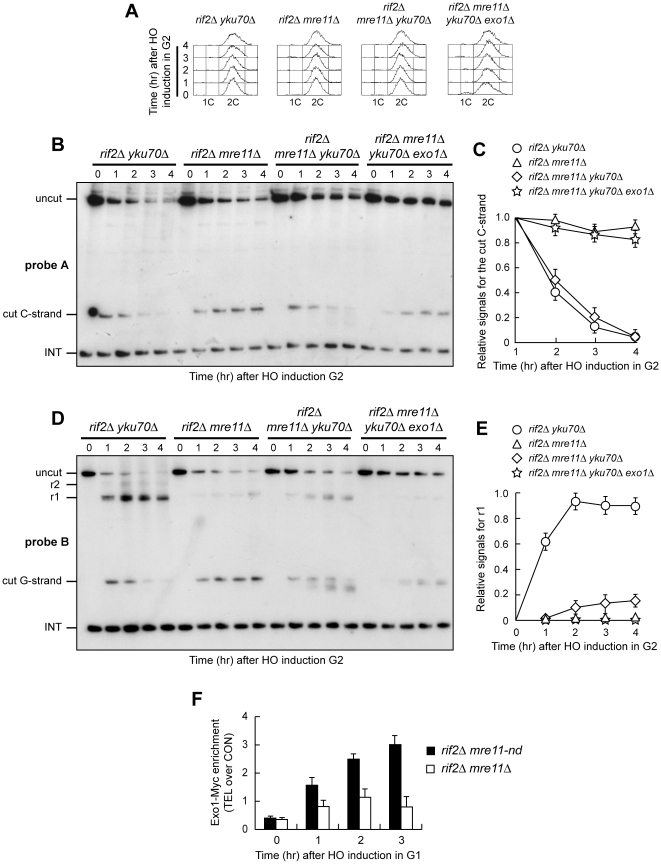
The lack of Yku allows Exo1-dependent resection in *rif2Δ* G2 cells lacking MRX. (A–E) HO expression was induced at time zero by galactose addition to nocodazole-arrested cells that were then kept arrested in G2. (A) FACS analysis of DNA content. (B) RsaI- and EcoRV-digested genomic DNA was hybridized with probe A as in Figure 1C. (C) Densitometric analysis. Plotted values are the mean value ±SD from three independent experiments as in (B). (D) The same RsaI- and EcoRV-digested genomic DNA analyzed in (B) was hybridized with probe B as described in Figure 1E. (E) Densitometric analysis. Plotted values are the mean value ±SD from three independent experiments as in (D). (F) HO expression was induced at time zero by galactose addition to α-factor-arrested *rif2Δ mre11-nd* and *rif2Δ mre11Δ* cells, all expressing a fully functional *EXO1-MYC* tagged allele. The cell cycle arrest in G1 was confirmed by FACS analysis (data not shown). Cells were then kept arrested in G1 and chromatin samples taken at different times after HO induction were immunoprecipitated with anti-Myc antibody. Coimmunoprecipitated DNA was analyzed by quantitative real-time PCR (qPCR) using primer pairs located at the nontelomeric *ARO1* fragment (CON) and 640 bp proximal to the HO site (TEL), respectively. Data are expressed as relative fold enrichment of TEL over CON signal after normalization to input signals for each primer set. The data presented are the mean of those obtained in three independent experiments. Error bars indicate s. d.

Yku-mediated inhibition of Exo1 is relieved in G2, as nucleolytic processing of an HO-induced telomere in G2-arrested *rif2Δ* cells depends on both Mre11 nuclease activity and Exo1 even when Yku is functional (Figure 3). However, unlike in *rif2Δ mre11-nd* G2 cells, 5′ C-strand resection did not occur in G2-arrested *rif2Δ mre11Δ* cells (Figure 3A and 3B). Similarly, the 5′ C-strand was not degraded in G1-arrested *rif2Δ mre11Δ* cells (Figure 1C and 1D), whereas a residual Exo1-dependent 5′ C-strand degradation was detectable in G1-arrested *rif2Δ mre11-nd* cells (Figure 2C and 2D). These observations suggest that Exo1-dependent resection in both G1 and G2 requires the presence of MRX, even if devoid of Mre11 nuclease activity. The lack of MRX was shown to increase the amount of Yku bound to DNA ends [49], [50], raising the possibility that the absence of MRX may enhance the inhibitory effect of Yku on Exo1. We then asked whether *YKU70* deletion allows Exo1-dependent resection in G1- and/or G2-arrested *rif2Δ mre11Δ* cells. Indeed, the 5′ C-strand, which remained stable in G1-arrested *rif2Δ mre11Δ* cells (Figure 1C and 1D), was degraded in *rif2Δ mre11Δ yku70Δ* G1 cells (Figure 6A and 6B). Similarly, 5′ C-strand degradation occurred in *rif2Δ mre11Δ yku70Δ* G2 cells, but not in *rif2Δ mre11Δ* G2 cells (Figure 7A–7C). In both cases, the observed nucleolytic degradation was dependent on Exo1, as it was abrogated in both G1-arrested (Figure 6A and 6B) and G2-arrested (Figure 7A–7C) *rif2Δ mre11Δ yku70Δ exo1Δ* cells. Thus, Yku appears to inhibit Exo1 not only in G1 but also in G2, although Yku-mediated Exo1 inhibition in G2 becomes apparent only in the absence of MRX.

The lack of Yku70 allowed Exo1-dependent resection in both G1- and G2-arrested *rif2Δ mre11Δ* cells (Figures 6 and 7), but this resection was confined to the telomeric tips. In fact, G1-arrested *rif2Δ mre11Δ yku70Δ* cells failed to convert the 5′ G-strand fragment into the r1 and r2 resection products, which instead accumulated in both *rif2Δ yku70Δ* and *rif2Δ yku70Δ exo1Δ* G1-arrested cells (Figure 6C and 6D). Similarly, r1 and r2 resection products accumulated in *rif2Δ yku70Δ* G2 cells, while they were only slightly detectable in *rif2Δ mre11Δ yku70Δ* G2 cells (Figure 7D and 7E). Thus, the absence of Yku in *rif2Δ mre11Δ* cells is not sufficient to ensure an efficient Exo1 function at the HO-induced telomere. In contrast, an Exo1-dependent resection takes place efficiently in *rif2Δ mre11-nd yku70Δ* G1 cells (Figure 4), suggesting that Exo1 likely requires the MRX complex in order to efficiently resect them.

One possibility is that the physical presence of MRX, even if defective for the nuclease activity, facilitates Exo1 recruitment onto DNA ends. Thus, we compared the binding of Exo1 at the HO-induced telomere in *rif2Δ mre11-nd* and *rif2Δ mre11Δ* cells. To minimize possible effects of resection on the binding of Exo1 to DNA ends, we examined association of Myc-tagged Exo1 by ChIP analysis at the HO-induced telomere in G1-arrested *rif2Δ mre11Δ* cells, which did not resect the 5′ C-strand, and in *rif2Δ mre11-nd* cells, where resection was severely compromised and did not proceed beyond 166 bp from the HO site (Figure 1E and 1F). Quantitative real-time polymerase chain reaction (qPCR) was then used to monitor coimmunoprecipitation with Exo1-Myc of a DNA fragment located 640 bp centromere-proximal to the HO site (TEL) and of a nontelomeric *ARO1* fragment (CON). As shown in Figure 7F, Exo1 association at the HO-induced telomere was reduced in *rif2Δ mre11Δ* compared to *rif2Δ mre11-nd* cells, supporting the hypothesis that the MRX complex promotes Exo1 association at DNA ends independently of it nuclease activity.

## Discussion

We previously showed that the lack of Rif2 or Yku allows resection of an HO-induced telomere in G1 cells [41]. Moreover, generation of ssDNA at both HO-induced and native telomeres in *rif2Δ* G1 cells is abolished in the absence of MRX, whereas Exo1 is responsible for nucleolytic degradation in *yku70Δ* G1 cells [41]. As MRX has functions in resection that are independent of Mre11 nuclease activity, we used the HO-induced telomere system to investigate both the nuclease activities that are regulated by Rif2 and how Rif2 and Yku functions are coordinated during the cell cycle. We found that resection of the HO-induced telomere in *rif2Δ* G1 cells depends primarily on Mre11 nuclease activity and Sae2, indicating that Exo1 and Sgs1 are not capable to bypass a defective Mre11 nuclease activity in this cell cycle phase. Because Sae2 has been shown to be a Cdk1 target in promoting ssDNA generation at DNA ends [17], [26], and Cdk1 activity is low in G1, this finding implies that the ability of Sae2 and MRX to resect DNA ends does not require Cdk1-dependent phosphorylation events. Notably, this Sae2- and MRX-dependent resection in G1 takes place only when the inhibitory function of Rif2 is abolished. This finding suggests that Cdk1-dependent phosphorylation events might simply potentiate the resection machinery by promoting the removal of negative regulators of DNA end resection.

The lack of Yku allows Exo1-dependent resection in *rif2Δ mre11-nd* G1 cells, indicating that Yku provides a block to resection by Exo1 in G1. We found that also Sgs1 contributes partially to 5′ C-strand resection in G1-arrested *rif2Δ mre11-nd yku70Δ* cells, but not in *rif2Δ mre11-nd* G1 cells, suggesting that Yku counteracts the function not only of Exo1, but also of Sgs1. Interestingly, Yku has a similar protective functions towards Sgs1 also at non telomeric DSBs, where it was recently shown to prevent Sgs1-dependent resection in the absence of functional MRX or Sae2 [51]. In any case, the Yku-mediated resection block is relieved in G2, where resection in *rif2Δ* cells depends on both Exo1 and Mre11 nuclease activity, while Sgs1 plays a minor role, if any.

Exo1 function appears to be counteracted also by Rif2, although this inhibition is detectable only in G2, where the Yku-mediated inhibition of Exo1 is relieved. This Rif2-mediated inhibition of Exo1 is in agreement with previous observations showing that accumulation of ssDNA at native telomeres after Rap1 inactivation is dependent on Exo1 in cycling but not in stationary phase cells [42]. Furthermore, it could explain why the contribution of Exo1 in DNA end resection is much more important than that of Sgs1 in *rif2Δ* G2 cells, whereas Exo1 and Sgs1 play redundant functions in resection in G2 cells carrying a functional Rif2 [26].

Unlike in G2-arrested *rif2Δ mre11-nd* cells, resection is undetectable in *rif2Δ mre11Δ* G2 cells. This finding suggests that neither Exo1 nor Sgs1 can resect DNA ends in G2 when MRX is absent, whereas they contribute to resection when MRX is bound to DNA ends but is nuclease-deficient. Similarly, the 5′ C-strand is not degraded in G1-arrested *rif2Δ mre11Δ* cells, whereas a residual Exo1-dependent 5′ C-strand degradation is detectable in G1-arrested *rif2Δ mre11-nd* cells. The lack of Yku is sufficient to initiate Exo1-dependent resection in both G1- and G2-arrested *rif2Δ mre11Δ* cells, indicating that Yku inhibits Exo1 not only in G1 but also in G2. However, while Yku-mediated inhibition of Exo1 in G1 occurs both in the presence and in the absence of MRX, it becomes apparent in G2 only when MRX is absent. Thus, removal of the Yku-mediated block to resection in G2 appears to require the physical presence of MRX, but not its nuclease activity, suggesting that Yku removal does not depend on the initial processing of the DSB ends by MRX-Sae2. As the lack of MRX leads to increased amounts of Yku associated to DNA ends [49], [50], it is tempting to suggest that MRX antagonizes Yku binding to DNA ends, thereby facilitating Exo1 recruitment and its associated resection activity. The same hypothesis was proposed recently also at non telomeric DSBs, where MRX was shown to facilitate Exo1 association to DSBs independently of Mre11 nuclease activity by promoting Yku removal [52]. In any case, while the physical presence of MRX is sufficient to relieve Yku-mediated inhibition of Exo1 in G2, it is required but not sufficient in G1, where resection in *rif2Δ* cells depends primarily on Mre11 nuclease activity. Because Cdk1 activity is high in G2, a likely possibility is that Cdk1-dependent phosphorylation events promote MRX-dependent removal of Yku in G2 by enhancing MRX-Sae2 access to DNA. However, we cannot exclude the possibility that Cdk1 contributes to MRX-dependent removal of Yku from DNA ends also by phosphorylating Yku and decreasing its ability to bind DNA ends. Both hypotheses are in agreement with the finding that Cdk1 requirement for HO-induced telomere end resection is bypassed when the inhibitory function of Yku is relieved.

We also provide evidence that, in addition to relieve Yku-mediated Exo1 inhibition, MRX is required to promote Exo1- and Sgs1-dependent resection independently of its nuclease activity. In fact, while resection in both G1- and G2-arrested *rif2Δ mre11Δ yku70Δ* cells does not depend on Sgs1, Sgs1 contributes to resect DNA ends in *rif2Δ mre11-nd yku70Δ* G1 cells. Thus, Sgs1 might need a structurally proficient MRX complex in order to exert its function in resection. In agreement with this hypothesis, MRX has been found in a complex with Sgs1 upon checkpoint activation [53] and stimulates Sgs1 activity in vitro [54], [55], suggesting that stimulation of resection by MRX might be due to Sgs1 recruitment to DNA ends. Furthermore, although Exo1 can initiate resection in both G1- and G2-arrested *rif2Δ mre11Δ* cells when Yku is absent, this resection is confined to the terminal part of the HO-induced telomere, while it is far reaching in *rif2Δ mre11-nd yku70Δ* cells. These observations suggest that the MRX complex bound to DNA ends might facilitate a stable Exo1-DNA association either directly or indirectly by stabilizing DNA ends. Consistent with this hypothesis, we found that cells lacking Mre11 show a reduced Exo1 association at the HO-induced telomere in G1 compared to cells carrying the nuclease-defective Mre11 variant.

**Figure 8:**
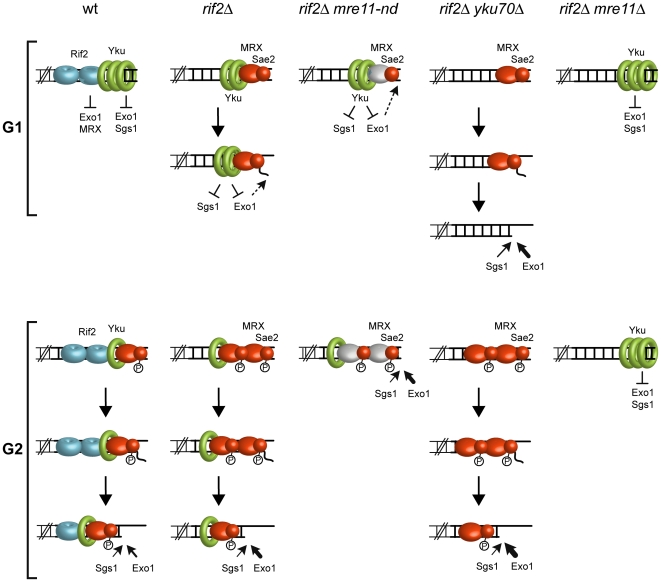
A working model for resection of DNA ends in G1 and G2. Rif2 inhibits both MRX and Exo1. In G1, the absence of Rif2 allows MRX recruitment to DNA ends to initiate nucleolytic resection of the 5′-strand. This resection depends primarily on MRX nuclease activity and Sae2, because Yku prevents both Exo1 and Sgs1 functions. Only Exo1 contributes to resection, although it does not bypass the requirement of Mre11 nuclease activity. The absence of Yku allows Exo1 and Sgs1 to compensate for a defective Mre11 nuclease activity, with Exo1 playing the major role. In G2, Cdk1 enhances Sae2/MRX function in resection by phosphorylating Sae2. The Yku-mediated block to Exo1 and Sgs1 is relieved and resection depends on MRX nuclease activity, Exo1 and, to a minor extent, Sgs1. In the absence of Rif2, Exo1 contribution in resection is increased compared to that of Sgs1. The Yku-mediated block is override even in *rif2Δ* cells defective for Mre11 nuclease activity, where resection depends on Exo1 and, to a minor extent, Sgs1. When MRX is not bound to DNA ends, resection does not take place in both G1 and G2 because Yku-mediated block is not relieved and Sgs1 function is also prevented by the lack of MRX. Even if Yku is absent in *mre11Δ* cells, Exo1 resects DNA ends inefficiently. Nuclease proficient MRX is in orange, while nuclease defective MRX is in grey.

Based on our data, MRX appears to play multiple functions in promoting DNA end resection independently of Mre11 nuclease activity: i) it promotes Yku removal, ii) it allows Sgs1-mediated resection and iii) it increases the efficiency of Exo1 activity. These findings can explain why the lack of MRX causes more severe resection defects and higher sensitivity to DNA damaging agents than *mre11* nuclease defective mutations [30], [33]. Altogether, our findings suggest a model for regulation of DNA end resection in G1 and G2 (Figure 8). In G1, Yku is bound to DNA ends and blocks access to Exo1 and Sgs1. When the Rif2 protective function is relieved, a Cdk1-independent resection takes place, which relies mainly on MRX nuclease activity. Exo1 and Sgs1 can compensate for defective MRX nuclease activity in G1 only if Yku is absent. When cells are in G2, Yku-mediated block is relieved and resection in *rif2Δ* cells depends on Mre11 nuclease activity, Exo1 and, to a minor extent, Sgs1. Yku removal in G2 requires MRX bound to DNA ends, but not Mre11 nuclease activity, suggesting that MRX and Yku compete for binding to DNA ends. Sae2 phosphorylation by Cdk1 in turn facilitates MRX-mediated removal of Yku. Finally, in addition to relieve Yku-mediated block to resection, MRX is also required to promote Exo1- and Sgs1-dependent resection, possibly by facilitating Exo1 and Sgs1 recruitment to DNA ends. Given the critical role of resection for telomere homeostasis, it will be an important issue for future research to determine whether these or similar mechanisms also act at native telomeres.

## Materials and Methods

### Yeast strains and media

Strain genotypes are listed in supplementary Table S1 online. The strains used for monitoring telomere resection at the HO-induced telomere were derivatives of strain UCC5913 kindly provided by D. Gottschling (Fred Hutchinson Cancer Research Center, USA). All gene disruptions were carried out by PCR-based methods. PCR one-step tagging was used to obtain strains carrying a fully functional MYC-tagged *EXO1* allele. In order to allow efficient and persistent G1 arrest, all strains carried the deletion of the *BAR1* gene, encoding a protease that degrades the mating pheromone α-factor. Cells were grown in YEP medium (1% yeast extract, 2% bactopeptone, 50 mg/l adenine) supplemented with 2% glucose (YEPD) or 2% raffinose (YEP+raf) or 2% raffinose and 2% galactose (YEP+raf+gal). All the experiments were carried out at the temperature of 26°C.

### Resection assay

To monitor resection at the HO-derived telomere, RsaI- and EcoRV-digested genomic DNA was subjected to denaturing polyacrilammide gel electrophoresis and then hybridized with the single-stranded riboprobes A or B, which anneal to the 5′ C-strand or the 3′ G-strand, respectively, to a site located 212 nt from the HO cutting site. For quantitative analysis of C-strand signals, the ratios between the intensities of C-strand and loading control bands were calculated by using the NIH image program. For quantitative analysis of r1 resection products signals, the ratios between the intensities of r1 resection product and loading control bands were calculated by using the NIH image program.

### ChIP analysis

ChIP analysis was performed as described [56]. After exposure to formaldehyde, chromatin samples were immunoprecipitated with anti-Myc antibody. Quantification of immunoprecipitated DNA was achieved by qPCR on a Biorad MiniOpticon using primer pairs located at the nontelomeric *ARO1* fragment of chromosome IV (CON) and 640 bp centromere-proximal to the HO cutting site (TEL) and normalized to input signal for each primer set; data are expressed as the fold enrichment of TEL over the amount of CON in the immunoprecipitates.

## Supporting Information

Table S1 *Saccharomyces cerevisiae* strains used in this study.
(0.05 MB DOC)
