# Supplementary material for: The MRX Complex Plays Multiple Functions in Resection of Yku- and Rif2-Protected DNA Ends
Source: PLoS One. 2010 Nov 30;5(11):e14142. doi: 10.1371/journal.pone.0014142 (PMC2994746; doi:10.1371/journal.pone.0014142)
Supplement: Table S1 — Saccharomyces cerevisiae strains used in this study. (0.05 MB DOC) [file pone.0014142.s001.doc]

# Supporting Information

**Table S1.** *Saccharomyces cerevisiae* strains used in this study.

| Strain | Relevant genotype | Source or Reference |
| --- | --- | --- |
| UCC5913 | *MATa-inc ade2-101 lys2-801 his3-Δ200 trp1-Δ63 ura3-52 leu2-Δ1::GAL1-HO-LEU2 VII-L::ADE2-TG(1-3)-HO site-LYS2* | 1 |
| YLL2599 | UCC5913 *bar1Δ::HPHMX* | This study |
| YLL2647 | UCC5913 *yku70Δ::URA3 rif2Δ::NATMX* *bar1Δ::HPHMX* | This study |
| YLL2650 | UCC5913 *rif2Δ::NATMX bar1Δ::HPHMX* | This study |
| YLL2667 | UCC5913 *sae2Δ::KANMX4 rif2Δ::NATMX bar1Δ::HPHMX* | This study |
| YLL2718 | UCC5913 *sgs1Δ::URA3* *rif2Δ::NATMX bar1Δ::HPHMX* | This study |
| YLL2725 | UCC5913 *rif2Δ::NATMX mre11Δ::KANMX4 bar1Δ::HPHMX* | This study |
| YLL2731 | UCC5913 *rif2Δ::NATMX exo1Δ::URA3 bar1Δ::HPHMX* | This study |
| YLL2752 | UCC5913 *mre11Δ::NATMX::mre11H125N::URA3 bar1Δ::HPHMX* | This study |
| YLL2781 | UCC5913 *mre11Δ::KANMX4 yku70Δ::URA3 rif2Δ::NATMX* *bar1Δ::HPHMX* | This study |
| YLL2784 | UCC5913 *exo1Δ::NATMX yku70Δ::URA3 rif2Δ::TRP1* *bar1Δ::HPHMX* | This study |
| YLL2802 | UCC5913 *mre11Δ::NATMX::mre11H125N::URA3 rif2Δ::TRP1 bar1Δ::HPHMX* | This study |
| YLL2803 | UCC5913 *exo1Δ::NATMX mre11Δ::KANMX4 yku70Δ::URA3 rif2Δ::NATMX* *bar1Δ::HPHMX* | This study |
| YLL2806 | UCC5913 *sgs1Δ::KANMX4 mre11Δ::NATMX::mre11H125N::URA3 rif2Δ::TRP1 bar1Δ::HPHMX* | This study |
| YLL2807 | UCC5913 *exo1Δ::KANMX4 mre11Δ::NATMX::mre11H125N::URA3 rif2Δ::TRP1 bar1Δ::HPHMX* | This study |
| YLL2809 | UCC5913 *exo1Δ::URA3 sgs1Δ::KANMX4 rif2Δ::NATMX* *bar1Δ::HPHMX* | This study |
| YLL2816 | UCC5913 *mre11Δ::NATMX::mre11H125N::URA3 sgs1Δ::HIS3 exo1Δ::KANMX4 rif2Δ::TRP1 bar1Δ::HPHMX* | This study |
| YLL2817 | UCC5913 *yku70Δ::HIS3 mre11Δ::NATMX::mre11H125N::URA3 rif2Δ::TRP1 bar1Δ::HPHMX* | This study |
| YLL2820 | UCC5913 *yku70Δ::HIS3 mre11Δ::NATMX::mre11H125N::URA3 rif2Δ::TRP1 exo1Δ::KANMX4 bar1Δ::HPHMX* | This study |
| YLL2821 | UCC5913 *yku70Δ::HIS3 mre11Δ::NATMX::mre11H125N::URA3 rif2Δ::TRP1 sgs1Δ::KANMX4 bar1Δ::HPHMX* | This study |
| YLL2846 | UCC5913 *mre11Δ::KANMX4::mre11H125N::URA3 rif2Δ::NATMX bar1Δ::HPHMX EXO1-18MYC* | This study |
| YLL2847 | UCC5913 *mre11Δ::KANMX4 rif2Δ::NATMX bar1Δ::HPHMX EXO1-18MYC* | This study |

**References**

1. Diede SJ, Gottschling DE (2001) Exonuclease activity is required for sequence addition and Cdc13p loading at a de novo telomere. Curr Biol 11: 1336-1340.
